# Effects of 2′,4′-Dihydroxy-6′-methoxy-3′,5′-dimethylchalcone from *Syzygium nervosum* Seeds on Antiproliferative, DNA Damage, Cell Cycle Arrest, and Apoptosis in Human Cervical Cancer Cell Lines

**DOI:** 10.3390/molecules27041154

**Published:** 2022-02-09

**Authors:** Kraikrit Utama, Nopawit Khamto, Puttinan Meepowpan, Paitoon Aobchey, Jiraporn Kantapan, Korawan Sringarm, Sittiruk Roytrakul, Padchanee Sangthong

**Affiliations:** 1 Interdisciplinary Program in Biotechnology, Graduate School, Chiang Mai University, Chiang Mai 50200, Thailand; kraikrit_uta@cmu.ac.th; 2 Department of Chemistry, Faculty of Science, Chiang Mai University, Chiang Mai 50200, Thailand; nopawit_kh@cmu.ac.th (N.K.); pmeepowpan@gmail.com (P.M.); 3 Research Center on Chemistry for Development of Health Promoting Products from Northern Resources, Faculty of Science, Chiang Mai University, Chiang Mai 50200, Thailand; 4 Science and Technology Research Institute, Chiang Mai University, Chiang Mai 50200, Thailand; paitoon.aob@cmu.ac.th; 5 Department of Radiologic Technology, Faculty of Associated Medical Sciences, Chiang Mai University, Chiang Mai 50200, Thailand; jiraporn.kan@cmu.ac.th; 6 Department of Animal and Aquatic Sciences, Faculty of Agriculture, Chiang Mai University, Chiang Mai 50200, Thailand; korawan.s@cmu.ac.th; 7 Functional Ingredients and Food Innovation Research Group, National Center for Genetic Engineering and Biotechnology, National Science and Technology Development Agency, Bangkok 12120, Thailand; sittiruk@biotec.or.th

**Keywords:** chalcone derivative, 2′,4′-dihydroxy-6′-methoxy-3′,5′-dimethylchalcone, *Syzygium nervosum*, antiproliferative cervical cancer activity, compound-induced apoptosis

## Abstract

2′,4′-Dihydroxy-6′-methoxy-3′,5′-dimethylchalcone (DMC), a natural product derived from *Syzygium nervosum* A. Cunn. ex DC., was investigated for its inhibitory activities against various cancer cell lines. In this work, we investigated the effects of DMC and available anticervical cancer drugs (5-fluorouracil, cisplatin, and doxorubicin) on three human cervical cancer cell lines (C-33A, HeLa, and SiHa). DMC displayed antiproliferative cervical cancer activity in C-33A, HeLa, and SiHa cells, with IC_50_ values of 15.76 ± 1.49, 10.05 ± 0.22, and 18.31 ± 3.10 µM, respectively. DMC presented higher antiproliferative cancer activity in HeLa cells; therefore, we further investigated DMC-induced apoptosis in this cell line, including DNA damage, cell cycle arrest, and apoptosis assays. As a potential anticancer agent, DMC treatment increased DNA damage in cancer cells, observed through fluorescence inverted microscopy and a comet assay. The cell cycle assay showed an increased number of cells in the G_0_/G_1_ phase following DMC treatment. Furthermore, DMC treatment-induced apoptosis cell death was approximately three- to four-fold higher compared to the untreated group. Here, DMC represented a compound-induced apoptosis for cell death in the HeLa cervical cancer cell line. Our findings suggest that DMC, a phytochemical agent, is a potential candidate for antiproliferative cervical cancer drug development.

## 1. Introduction

The global incidence of cancer is continually increasing, with an estimated 18.1 million new patients diagnosed with cancer in 2018 [1,2]. Cervical cancer accounts for an estimated 65,620 new cases and 12,590 deaths per year [3]. In 2020, incidence and mortality rates of cervical cancer have been increased in most regions of the world. On the other hand, the frequency of cervical cancer is increasing in developing countries [4]. Cervical cancer treatments are currently as follows: chemotherapy, surgery, and radiation therapy, which depend on early (IA-IB1) and advanced (IB2-IVB) stage. However, the limitations of cancer treatment are long-term recovery after surgery and/or radiation therapy as well as damage to normal cells after treatment with chemotherapy [5,6]. Anticancer drugs (such as 5-fluorouracil, cisplatin, and doxorubicin) are available for cervical cancer treatment; however, these drugs have several adverse side effects, such as irreversible cardiotoxicity, reversible nephrotoxicity, gastrointestinal symptoms, neurotoxicity, nephrotoxicity, and ototoxicity [7,8,9]. Toxicity associated with several chemotherapeutic approaches has raised the need for the elucidation and development of more anticancer drugs. Several studies have reported the efficacy of natural phytochemicals found in medicinal plants and fruits, and they have recently been investigated as potential anticancer agents and in the development of anticancer drugs. *Syzygium nervosum* A. Cunn. ex DC or *Cleistocalyx nervosum*, locally named Ma-kiang, is found in Southeast Asia: Vietnam, China, and Thailand. It belongs to the Myrtaceae family. The decoction of leaves and flower buds from *S. nervosum* have long been consumed in the Vietnamese and Chinese communities. In addition, *S. nervosum* has been used in traditional medicine where it is purposed for influenza, cancer, skin diseases, and digestive conditions [10] Currently, the chemical profiling and pharmacological activities of *S. nervosum* crude extract and isolated pure compounds have been widely published. For example, the main biological activities of *S. nervosum* seed extract include anticancer and antigenotoxic activities. The seed’s methanolic extract was found to inhibit aflatoxin B1 and MeIQ (2-amino-3,4-dimethylimidazo-[4,5-*f*]quinolone)-induced mutagenesis in a dose-dependent manner in a *Salmonella* mutation assay. This effect might occur through the inhibitory effects on carcinogen-bioactivated enzymes rather than a direct action on mutagens [10,11]. The main chemical compound isolated from the buds and seeds of *S. nervosum* is 2′,4′-dihydroxy-6′-methoxy-3′,5′-dimethylchalcone (DMC), which has been reported for biological activities, including anti-inflammatory, antiviral, antidiabetic, and anticancer [12,13,14,15,16].

**Figure 1:**
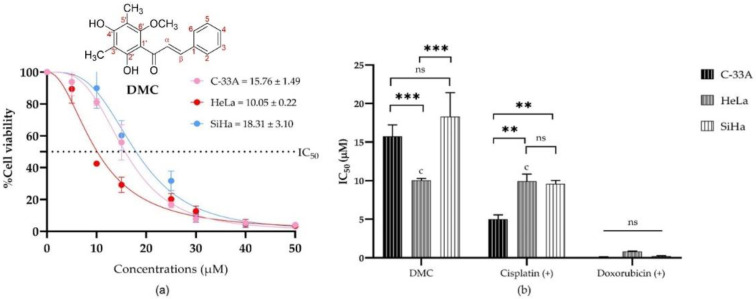
The half maximal inhibitory concentrations (IC_50_) of DMC in human cervical cancer cell lines (C-33A, HeLa, and SiHa): The percentage cell viability of C-33A, HeLa, and SiHa human cervical cancer cell lines treated with different concentrations of DMC (0–50 µM) (**a**). The IC_50_ of DMC and the available anticervical cancer drugs compression on anticervical cancer activities (**b**). Significant differences are shown at ** *p* ≤ 0.02, and *** *p* ≤ 0.001; ns, not significant (*p* ≥ 0.05).

In northern Thailand, Ma-kiang is used in various food products including juices, food processing, and health food products, and appears in agricultural waste [17]. Alternatively, natural products from agricultural waste can be used in pharmacological applications, such as the production of anticancer drugs and anticancer developments for high value-added products [15]. In addition, the main pure compound from the seeds of *S. nervosum* from agricultural waste is DMC [15,16]. The chemical structure of DMC has been elucidated, as shown in Figure 1 [15,16,17,18,19,20]. DMC is a chalcone derivative that has many reported biological activities, including anti-inflammatory, antiviral, antidiabetic, and anticancer [12,13,14,15]. In vitro anticancer activities of DMC have been demonstrated in leukemia, colorectal, liver, breast, and pancreatic cancers [15,21,22,23]. In a previous study, the mechanisms of DMC-induced cell apoptosis, cell death, and G_0_/G_1_ cell cycle arrest were found to involve the PI3K/AKT mitochondria-dependent pathway and increased generation of reactive oxygen species (ROS) in hepatocellular carcinoma (BEL-7402 and BEL-7402/5-FU). In addition, DMC shows low toxicity in human normal liver (L-02) cells and human normal fetal lung fibroblast cells (HFL-1), as well as in an ICR mouse model [24,25]. However, the anticervical cancer activity of DMC has not yet been fully investigated. This research aimed to investigate the antiproliferative cervical cancer activities of DMC. Potential antiproliferative cervical cancer agents are investigated for their DMC-induced apoptosis, including DNA damage, cell cycle arrest, and apoptosis assays, to confirm the possible mechanisms of antiproliferative cervical activity. This information could provide a better understanding of the antiproliferative cancer pathways in an in vitro model.

## 2. Results

### 2.1. Isolation and Structural Identification of DMC

DMC was isolated from *S. nervosum* seeds by maceration using CH_2_Cl_2_ as the solvent and purified using a chromatographic technique. TLC chromatogram of crude CH_2_Cl_2_ extract from *S. nervosum* seeds visualized under UV 254 nm (Figure S2). The chemical structure of DMC was verified by comparison of its spectroscopic data to previously published values, and all values were found to be identical with previous study [15,16]. The physical appearance of DMC is a yellow needle-shaped crystals obtained from crystallization using *n*-hexane-CH_2_Cl_2_, and its melting point is 124.2–125.6 °C. The UV–VIS spectrum of DMC in phosphate buffered saline at pH 7.4 (Figure S3) showed and minor band of benzoyl system at wavelength of maximum absorbance (λ_max_) of 228 nm and major band of cinnamoyl system at λ_max_ of 395 nm, which are characteristic of flavonoids with a structural skeleton of chalcone [26]. The methoxy group (O-CH_3_) showed absorption band at λ_max_ of 314 nm [27].

The FTIR spectrum showed an O-H stretching absorption band of free or chelated hydroxy groups at 3306 cm^−1^ and C=O stretching of an α,β-unsaturated ketone at 1630 cm^−1^, which is characteristic of a conjugated ketone (Figure S3). The ^1^H-NMR spectrum showed resonance signals of the monosubstituted aromatic (ArCH) system as multiplet in the range of *δ* 7.37–7.44 and 7.61–7.67 ppm (Figure S4). In addition, α,β-unsaturated protons resonated at *δ* 7.84 (β-CH) and 7.99 (α-CH) ppm as doublet in AB system coupling pattern, with *J* = 15.7 Hz characterized as a *trans*-double bond, the methoxy group (6′-OCH_3_) resonated at *δ* 3.66 ppm as singlet, the two methyl groups at *δ* 2.13 (3′-CH_3_) and 2.15 (5′-CH_3_) ppm as singlet, and the two hydroxy group resonated at *δ* 5.50 (4′-OH) and 13.62 (2′-OH) ppm, assigned as free and chelated hydroxy groups, respectively (Figure S4). ^13^C-NMR spectrum compared with DEPT90 and DEPT135 spectra indicated signals of two aromatic methyl carbons resonated at *δ* 7.7 (5′-CH_3_) and 8.4 (3′-CH_3_) ppm. Methoxy carbons (6′-OCH_3_) resonated at *δ* 62.5 ppm. Moreover, carbons signals of monosubstituted aromatic ring were observed at *δ* 128.6 (3,5-ArCH), 129.1 (2,6-ArCH), 130.4 (4-ArCH), and 135.5 (1-ArC) ppm. Fully substituted aromatic ring showed the signals of carbons resonated at *δ* 106.7 (1′-C), 109.1 (3′-C), 109.2 (5′-C), 159.0 (6′-C), 159.4 (4′-C), and 162.2 (2′-C) ppm. α,β-unsaturated carbons were observed at *δ* 126.8 (α-CH) and 143.1 (β-CH) ppm (Figure S5). Finally, the carbonyl group (C=O) was resonated at *δ* 193.5 ppm, which was high field than common ketone as characteristic of α,β-unsaturated carbonyl group. The structure was further confirmed by analyzing 2D-NMR spectra including ^1^H-^1^H Correlation spectroscopy (COSY), heteronuclear single-quantum coherence (HSQC), and heteronuclear multiple-bond correlation (HMBC) (see supporting information Figures S6–S8 and Table S1). All spectroscopic data resembled the previously reported data in the literature [15].

### 2.2. Antiproliferative Activity of DMC on Cervical Cancer Cell Lines

The antiproliferative activities of DMC and the anticancer drugs (5-fluorouracil, cisplatin, and doxorubicin) were investigated by MTT assay. The IC_50_ of DMC and the available anticancer drugs are shown in Table 1. DMC showed potential antiproliferative cervical cancer activity, with IC_50_ values of 10.05 ± 0.22, 15.76 ± 1.49, and 18.31 ± 3.10 µM in HeLa, C-33A, and SiHa cells, respectively (Table 1 and Figure 1a). Similarly, cisplatin treatment in HeLa cells had an IC_50_ value of 9.93 ± 0.16 µM. The IC_50_ values of DMC, cisplatin and doxorubicin were significantly different, with *p*-values of *p* ≤ 0.033, *p* ≤ 0.02, and *p* ≤ 0.001, respectively, compared to every other cell model in the two-way ANOVA (Figure 1b). Thus, DMC represents a promising specific antiproliferative cervical cancer agent in HeLa cells.

### 2.3. Investigation of Compound-Induced Apoptosis

However, DMC has not yet been fully studied for its biological properties in cervical cancer. Accordingly, DMC has drawn attention for further investigation as a promising anticancer drug for cervical cancer Thus, DMC induces apoptosis were investigated by comet, cell cycle, and apoptosis assays and compared to the three currently available anticervical cancer drugs.

#### 2.3.1. DMC-Induced DNA Damage Pathways

The effect of treatment with DMC as an antiproliferative cervical cancer drug candidate on DNA damage pathways was investigated by comet assay and compared to untreated and 1% DMSO groups [28,29]. The currently available anticervical cancer drugs, including 5-fluorouracil, cisplatin, and doxorubicin, were used as positive controls. The comet assay was performed with HeLa cells treated with DMC, the available anticervical cancer drugs, 1% DMSO, or untreated cells for specific time periods (6, 12, 24, and 48 h) under alkaline conditions. Comet assay with alkaline conditions can explain the single- and double-strand break with DNA damage and DNA repair after treatment with anticancer drugs [30,31,32].

**Figure 2:**
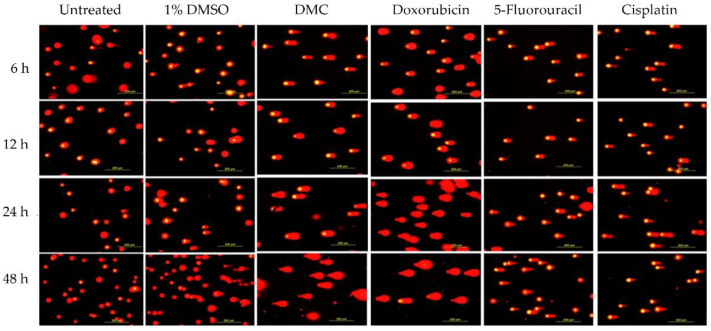
Compound-induced DNA damage pathways in HeLa human cervical cancer cells treated with DMC and standard anticervical cancer drugs when compared to untreated cells and cells treated with 1% DMSO for 6, 12, 24, and 48 h, as assessed by comet assay.

**Figure 3:**
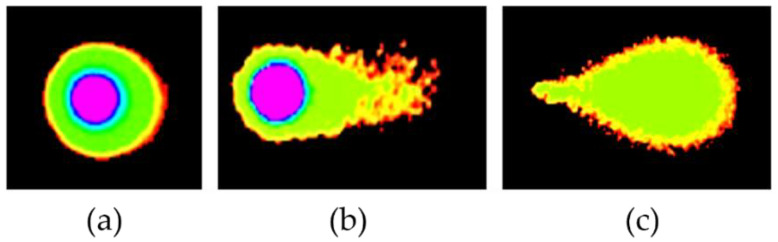
Compound-induced DNA damage patterns in HeLa human cervical cancer cells treated with DMC when compared with standard anticancer drugs, as analyzed using Comet Score version 2.0 software. Untreated pattern (**a**) and DNA damage patterns (**b**,**c**).

**Figure 4:**
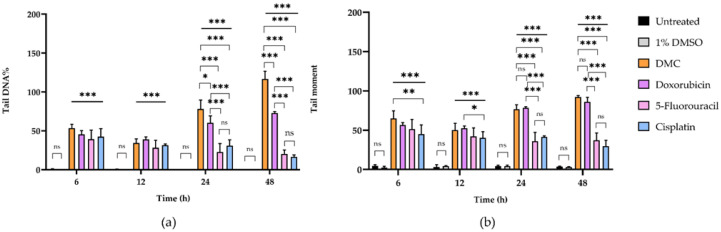
The DNA damage parameters of HeLa human cervical cancer cells after treatment with DMC and standard anticancer drugs compared to untreated cells and cells treated with 1% DMSO for 6, 12, 24, and 48 h, including tail DNA% (**a**) and tail moment (**b**). Significant differences are shown at * *p* ≤ 0.033, ** *p* ≤ 0.02, and *** *p* ≤ 0.001; ns, not significant (*p* ≥ 0.05).

The DNA tails of HeLa cells after treatment with DMC for 6, 12, 24, and 48 h showed compound-induced DNA damage patterns. Similar results were observed in cells treated with the available anticervical cancer drugs for 6, 12, 24, and 48 h when compared to the 1% DMSO and untreated groups, as shown in Figure 2 and Figure 3. Images of DNA tails were analyzed by Comet Score version 2.0, with a selected sample size (n) of 50. The analyzed parameters, including the tail moment and tail DNA%, are presented in Table 2. The DNA damage parameters in HeLa cervical cancer cells after treatment with DMC and the available anticervical cancer drugs demonstrated significant differences at * *p* ≤ 0.033, ** *p* ≤ 0.02, and *** *p* ≤ 0.001, as indicated in Figure 4, when compared to untreated and 1% DMSO groups. After treatment with DMC for 6, 12, 24, and 48 h, the tail DNA% of HeLa cells increased 14-, 15-, 17-, and 25-fold, respectively, compared to the untreated group. In addition, tail DNA% in the DMC group was 27-, 11-, 18-, and 30-fold higher than the 1% (w/w) DMSO group at 6, 12, 24, and 48 h, respectively. The tail moment values in cells treated with DMC were increased by 102-, 107-, 313-, and 833-fold compared to the untreated group at 6, 12, 24, and 48 h, respectively. Moreover, comparison of the percentage tail moment between DMC and 1% (w/w) DMSO groups showed 381-, 143-, 355-, and 1296-fold increases after 6, 12, 24, and 48 h of treatment, respectively. These results demonstrate increased DNA damage after treatment with DMC for the various incubation times. In addition, the tail DNA% and tail moment values of HeLa cells after treatment with DMC were similar to those observed after doxorubicin treatment. However, HeLa cells treated with 5-fluorouracil and cisplatin showed an increase in tail moment and tail DNA% after the first 6 h of treatment, but these values subsequently decreased at 12, 24, and 48 h.

#### 2.3.2. Inhibition of the G_0_/G_1_ and G_2_/M Phases

**Figure 5:**
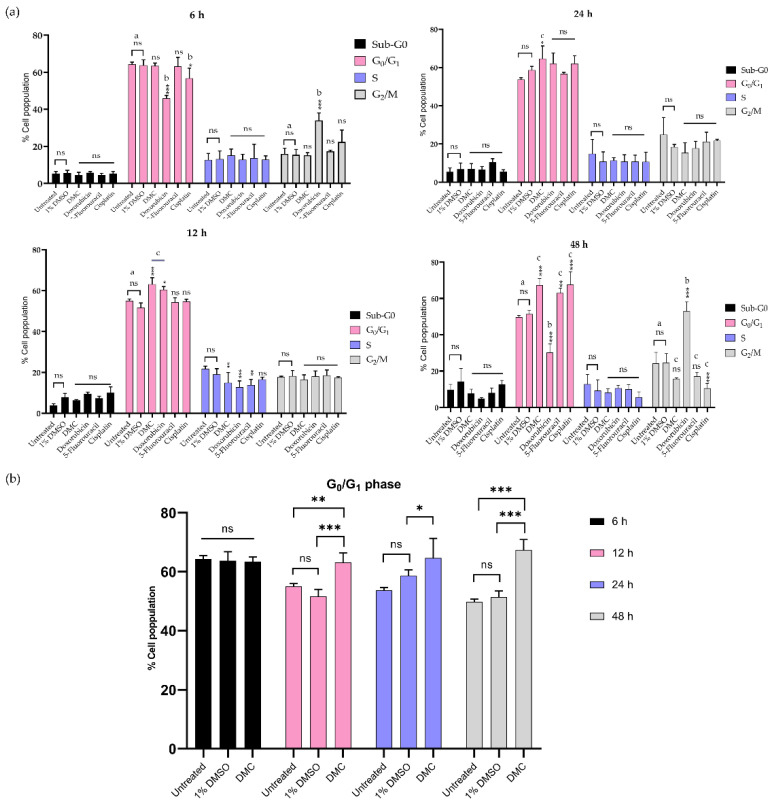
Treatment with DMC when compared to standard anticancer drugs induced cell cycle block in HeLa cells in vitro for 6–48 h after treatment (**a**). The percentage of HeLa cells in the G_0_/G_1_ phase after treatment with DMC for 6–48 h (**b**). Significant differences are shown at * *p* ≤ 0.033, ** *p* ≤ 0.02, and *** *p* ≤ 0.001; ns, not significant (*p* ≥ 0.05). ^a,b,c^ Classification groups based on the biological activity in Tukey’s model of two-way ANOVA.

This work investigated the mechanism of DMC-induced cell death through the induction of cell cycle arrest after treatment with DMC at its IC_50_ concentration in a time-dependent manner (6, 12, 24, and 48 h). HeLa cells treated with DMC, the available anticervical cancer drugs, or 1% (w/w) DMSO were analyzed by flow cytometry, and the results are presented in Figure 5 and Figure S9. The results showed an increased population of cells in the G_0_/G_1_ phase after treatment with DMC, estimated at 63.13 ± 3.21, 64.67 ± 6.66, and 67.40 ± 3.51 after 12, 24, and 48 h, respectively, when compared to untreated cells and cells treated with 1% DMSO. After 6 h, HeLa cells treated with DMC showed no significant differences when compared to untreated cells and cells treated with 1% DMSO. In addition, the effect of DMC on the G_0_/G_1_ phase of HeLa cells was similar to the effect after 12 h of doxorubicin treatment. In addition, the findings were similar to those observed for HeLa cells treated with 5-fluorouracil and cisplatin for 48 h. The G_0_/G_1_ and G_2_/M phase values of HeLa cells treated with DMC and the available anticervical cancer drugs were significantly different to the untreated and 1% DMSO groups (* *p* ≤ 0.033, ** *p* ≤ 0.02, and *** *p* ≤ 0.001) in the two-way ANOVA. Non-significant (ns) values had a *p*-value ≥ 0.12. Accordingly, DMC treatment demonstrated potential antiproliferative cervical cancer activities in HeLa cells by inhibiting proliferation in the G_0_/G_1_ phase of the cell cycle.

#### 2.3.3. DMC-Induced Apoptosis

**Figure 6:**
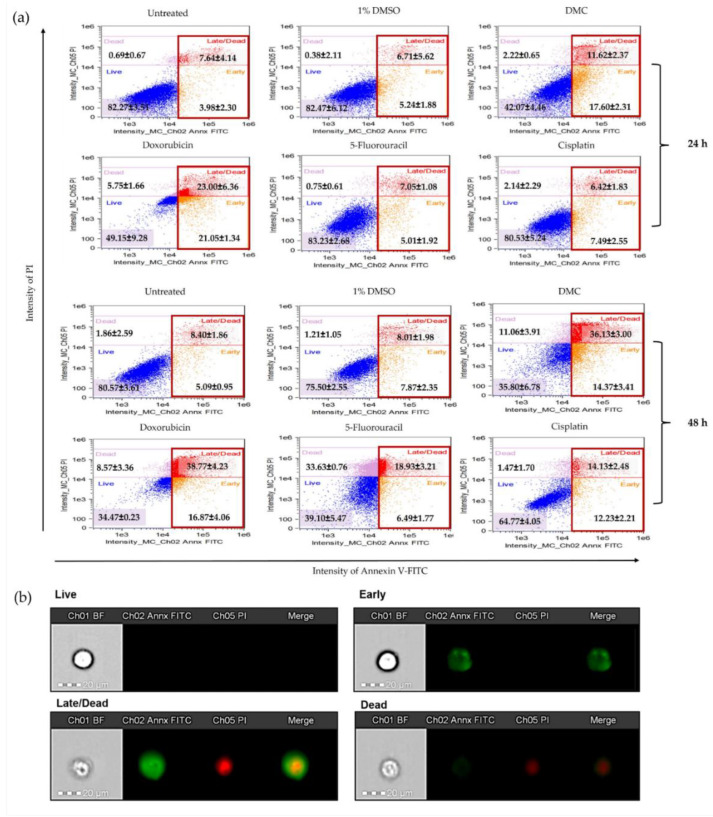
Analysis of apoptosis of HeLa cells after treatment with DMC when compared to standard anticancer drugs, as indicated by annexin V-fluorescein isothiocyanate (FITC) and propidium iodide (PI) staining (**a**). Morphology of HeLa cell populations after treatment with DMC and available agents for 24 and 48 h, stained with annexin V-FITC and PI and assessed using flow cytometry (**b**).

**Figure 7:**
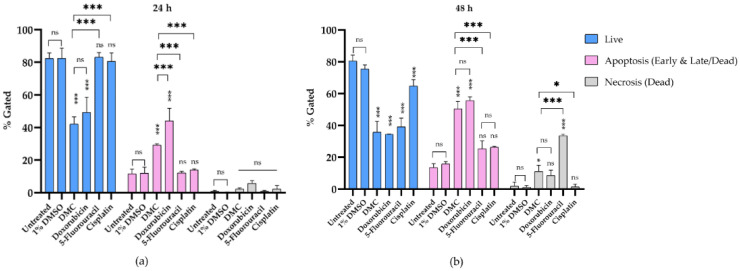
The percentage of apoptosis cell death in HeLa cells after treatment with DMC when compared to standard anticancer drugs for 24 h (**a**) and 48 h (**b**). Significant differences are shown at * *p* ≤ 0.033, and *** *p* ≤ 0.001; ns, not significant (*p* ≥ 0.05).

HeLa cells treated with DMC and anticervical cancer agents at IC_50_ concentrations for 24 and 48 h clearly showed DNA damage and inhibition of proliferation in the cell cycle. Therefore, we investigated the apoptosis pathway by flow cytometry, and the results are presented in Figure 6a. HeLa cells treated with DMC, the available anticervical cancer drugs, or 1% (w/w) DMSO were evaluated for annexin V-FITC and PI staining. After treatment with the active compounds, HeLa cells were categorized into four populations: live, early, late/dead, and dead populations (Figure 6b). The live population did not show any annexin V-FITC or PI staining. In the early population, only annexin V-FITC staining was detected. The late/dead population presented both annexin V-FITC and PI staining. Finally, the dead population showed only PI staining. The early and late/dead populations were involved in the apoptosis pathway. We then investigated the percentage of apoptosis cell death after treatment with DMC and the three anticervical cancer agents. The percentage of apoptosis cell death increased after treatment with DMC, estimated to be 29.22 ± 0.73% and 50.50 ± 4.58% after 24 and 48 h, respectively, when compared to the untreated and 1% DMSO groups. Similarly, doxorubicin treatment of HeLa cells for 48 h showed approximately 55.63 ± 2.35% apoptosis cell death. Interestingly, treatment of HeLa cells with doxorubicin for 24 h showed an increased percentage of apoptosis cell death compared to DMC treatment, with an estimated increase of 44.05 ± 7.71%, which is twofold higher than the cell death observed following DMC treatment. In addition, cisplatin and 5-fluorouracil treatment of HeLa cells for 48 h showed an estimated 25.42 ± 4.93% and 26.36 ± 0.60% apoptosis cell death, respectively. Treatment with cisplatin and 5-fluorouracil for 24 h did not increase the percentage apoptotic cell death of HeLa cells compared to the untreated and 1% DMSO groups. The percentage of apoptosis cell death observed in HeLa cells treated with DMC and the available anticervical cancer drugs demonstrated significant differences T * *p* ≤ 0.033, ** *p* ≤ 0.02, and *** *p* ≤ 0.001 when compared to the untreated and 1% DMSO groups in the two-way ANOVA. Non-significant (ns) differences showed *p*-values ≥ 0.12, as shown in Figure 7. Moreover, the death populations were considered to be an indicator of inflammatory mediators after treatment with DMC and the available anticervical cancer drugs [33]. DMC treatment of HeLa cells for 24 h showed a low percentage of inflammatory mediators, estimated to be 2.22 ± 0.65%, which was similar to that observed for doxorubicin treatment. After 48 h, HeLa cells treated with DMC showed a low percentage of inflammatory mediators (11.06 ± 3.91%), when compared to cells treated with 5-fluorouracil (33.63 ± 0.76%), similar to that observed for doxorubicin treatment. Therefore, DMC treatment demonstrated potential antiproliferative cervical cancer activity in HeLa cells involving apoptosis pathways.

## 3. Discussion

The majority of publications on *Syzygium nervosum* A. Cunn. ex DC. have focused on its phytochemical content, such as chalcone and flavanone derivatives [10,15,16]. DMC is a chalcone derivative isolated from the seeds of *S. nervosum*. DMC presents many biological activities, such as antioxidant, antiviral, and anticancer activities [32,33,34]. However, DMC has not been fully investigated for its effect on cell damage in cervical cancer. In our work, we investigated the biological activities of DMC from *S. nervosum*. Accordingly, DMC showed specific antiproliferative cervical cancer activities, with an IC_50_ value of 10.05 ± 0.22 µM in an adenocarcinoma model (HeLa), which was greater than the IC_50_ values for C-33A and SiHa cells. Similarly, cisplatin treatment showed an IC_50_ value of 9.93 ± 0.16 µM in HeLa cells. Previously, DMC has been shown a role in compound-induced apoptosis and G_0_/G_1_ cell cycle arrest through the PI3K/AKT mitochondria-dependent pathway and increased generation of reactive oxygen species (ROS) as part of the apoptosis pathways in hepatocellular carcinoma (BEL-7402 and BEL-7402/5-FU cell lines). Moreover, DMC was found to be nontoxic to human normal liver cells (L-02), human normal fetal lung fibroblast cells (HFL-1), and an ICR mouse model [24,25]. Therefore, in the current study, we investigated the biological properties of DMC as a potential antiproliferative cervical cancer agent using comet, cell cycle, and apoptosis assays.

After treatment with DMC for 6–48 h, HeLa cells showed increased DNA damage when compared to the untreated and 1% DMSO groups, similar to that observed for the available anticancer drugs. The increased tail moment and tail DNA percentage indicates DNA damage- and replication stress-induced double-strand and single-strand DNA breaks via ATM (Ataxia telangiectasia mutated)—and ATR (Ataxia telangiectasia and Rad3-related protein)—mediated pathways. The ATM and ATR signalling pathways are amplified by DNA damage signals, which activate the tumor suppressor p53 protein (p53) through the Checkpoint kinase 1 (CHK1) and Checkpoint kinase 2 (CHK2) regulator proteins, leading to cell cycle arrest and apoptosis [35]. Moreover, there was a decrease in tail moment and tail DNA percentage after treatment with cisplatin and 5-fluorouracil for 12, 24, and 48 h, indicating DNA repair. Similar to previously reports, 5-fluorouracil- and cisplatin-induced DNA damage primarily occurs through the DNA repair mechanism by nucleotide excision repair (NER) and DNA mismatch repair (MMR) pathways, which are involved in cisplatin resistance in human cervical cancer [36,37,38].

In addition, the proliferation of HeLa cells after treatment with DMC was time-dependent, as observed by flow cytometry. After 6 h of treatment with DMC, HeLa cells showed no change in the number of cells in the G_0_/G_1_ and G_2_/M phases. However, after 12–48 h of DMC treatment, the number of HeLa cells in the G_0_/G_1_ phase was increased, which indicates the inhibition of CDK/cyclin complexes by p16 and p21 following DNA damage and p53 activation, similar to that observed after 5-fluorouracil and cisplatin treatment for 48 h [36,37,38,39].

Moreover, HeLa cells treated with DMC for 24 and 48 h were evaluated for the percentage of apoptotic cell death by flow cytometry. After DMC treatment for 24 h, the percentage apoptosis cell death of HeLa cells was increased approximately threefold when compared to untreated cells and cells treated with 1% DMSO. Cells treated with 5-fluorouracil and cisplatin did not show any percentage of apoptosis cells death. On the other hand, doxorubicin increased the percentage of apoptosis cell death fourfold when compared to the untreated group. After 48 h of DMC treatment, HeLa cells demonstrated an estimated percentage of apoptosis cell death that was increased by fourfold when compared to the untreated and 1% DMSO groups, similar to that observed for doxorubicin treatment. DMC also presented higher potential antiproliferative cervical cancer activities, estimated to be twofold higher compared to cells treated with 5-fluorouracil or cisplatin. In addition, HeLa cells treated with DMC showed low inflammatory mediators, estimated to be threefold lower than that observed for 5-fluorouracil as an anticervical cancer drug. Inflammatory mediators can promote tumor growth and metastasis through the stimulation of cell proliferation and toxicity to healthy cells [40].

After treatment with DMC, HeLa cells showed increasing DNA damage, G_0_/G_1_ phases, and induced apoptosis cell death when compared to the untreated and 1% DMSO groups. These results suggest that the cell death mechanism by the ATM and ATR signalling pathways after increased DNA damage activates the p53 protein through the CHK1 and CHK2 regulator proteins, leading to cell cycle arrest and induced apoptosis cell death [41,42,43]. Accordingly, the findings of the current study suggest an antiproliferative cervical cancer effect of DMC through the induction of the cell death mechanism.

## 4. Materials and Methods

### 4.1. General Experimental Procedures for Structural Characterisation

Melting points were determined on a Gallenkamp Electrothermal apparatus (SANYO Gallenkamp, Leicestershire, UK). UV–VIS spectra were recorded on a UV–VIS spectrophotometer (Thermo Fisher Scientific Inc., Waltham, MA, USA). FTIR spectra were recorded on a Bruker Tensor 27 spectrometer (Bruker, Karlsruhe, Germany) and wavenumbers of maximum absorption peaks are reported in reciprocal centimeter (cm^−1^). ^1^H-NMR and ^13^C-NMR spectra were recorded on a Bruker Ascend^TM^ 500 spectrometer (Bruker, Karlsruhe, Germany) using CDCl_3_ as the solvent. 2D-NMR experiments including COSY, HSQC, and HMBC were carried for structural confirmation of DMC. Chemical shifts (*δ*) were reported as parts per million (ppm) downfield from tetramethylsilane (TMS) as an internal reference or using the residue solvent signal as an internal standard (CDCl_3_, *δ* 7.26 and 77.16 ppm for ^1^H-NMR and ^13^C-NMR, respectively) and coupling constants (*J* values) were reported in hertz (Hz). Peak multiplicities are indicated as follows: s (singlet), d (doublet), and m (multiplet). Column chromatography was performed using Merck silica gel 60 H (Merck KGaA, Darmstadt, Germany). Thin layer chromatography (TLC) was performed with Merck silica gel 60 F_254_ aluminum plates (Merck KGaA, Darmstadt, Germany).

### 4.2. Standard Anti-Cancer Drugs

Anticancer drugs contained doxorubicin hydrochloride injection USP ADRIM^®^ (Fresenius Kabi Oncology Ltd., Gurgaon, India), cisplatin Injection KEMOPLAT^®^ (Fresenius Kabi Oncology Ltd., India), and 5-fluorouracil or EFFCIL Injection 50 mg/10 mL (Boryung pharmaceutical Co., Ltd., Seoul, Korea).

### 4.3. Cell Culture

Human cervical cancer cell lines including C-33A (ATCC^®^ HTB-31™), HeLa (ATCC^®^ CCL-2™), and SiHa (ATCC^®^ HTB-35™) were obtained from the American Type Culture Collection. Human cervical cancer cell lines (C-33A, HeLa, and SiHa) were cultured in Eagle’s Minimum Essential Medium (EMEM) containing 10% fetal bovine serum (FBS) and 1% penicillin-streptomycin (10,000 U/mL). The cell lines were cultured and incubated at 37 °C in 5% CO_2_ atmosphere.

### 4.4. Isolation and Structural Identification of 2′,4′-Dihydroxy-6′-methoxy-3′,5′-dimethylchalcone

The isolation of DMC was achieved as previously reported [15,16]. The voucher specimen of the plant (BKF no. 187213) was deposited at the Forest Herbarium, Department of National Parks, Wildlife, and Plant Conservation, Ministry of Natural Resources and Environment, Bangkok, Thailand. Briefly, 5 kg of dried seed powder of *S. nervosum* was macerated with 10 L of CH_2_Cl_2_ (performed three times, each time for 3 days). The seed residues were filtered out, and the filtrate was collected and further concentrated under reduced pressure to produce crude CH_2_Cl_2_ extract as a dark green viscous liquid using a rotary evaporator (Hei-Vap Value Digital, Heidolph Instruments GmbH & Co., Schwabach, Germany). The extract was subjected to flash column chromatography on a silica gel, eluted with gradient concentrations of *n*-hexane-EtOAc from 100:0 to 80:20. The DMC-containing fractions were collected and further purified by crystallization with CH_2_Cl_2_–*n*-hexane to yield 2.8645 g of DMC as yellow needle-shaped crystals with high purity. The isolated DMC was verified by comparison to previously reported ^1^H- and ^13^C-NMR and FTIR spectra, which were identical to the published values.

DMC: yellow needle-shaped crystal; mp 124.2–125.6 °C; *R*_f_ (20%EtOAc/*n*-hexane) 0.45; FTIR (ATR) *ν*_max_ 3306 (OH), 3087, 3031 (=C-H), 2977, 2945, 2866 (CH_3_), 1630 (C=O), 1607, 1547 (C=C), 1229, 1166, 1115 (C-O) cm^−^^1^; ^1^H-NMR (CDCl_3_, 500 MHz): *δ* 2.13 (3H, s, 3′-CH_3_), 2.15 (3H, s, 5′-CH_3_), 3.66 (3H, s, 6′-OCH_3_), 5.50 (1H, s, 4′-OH), 7.37–7.44 (3H, m, ArCH), 7.61–7.67 (2H, m, ArCH), 7.84 (1H, AB system, d, *J* = 15.7 Hz, α-CH), 7.99 (1H, AB system, d, *J* = 15.7 Hz, β-CH), 13.62 (1H, s, 2′-OH); ^13^C-NMR (CDCl_3_, 125 MHz): 7.7 (5′-CH_3_), 8.4 (3′-CH_3_), 62.5 (6′-OCH_3_), 106.7 (1′-C), 109.1 (3′-C), 109.2 (5′-C), 126.8 (α-CH), 128.6 (3,5-CH), 129.1 (2,6-CH), 130.4 (4-CH), 135.5 (1-C), 143.1 (β-CH), 159.0 (6′-C), 159.4 (4′-C), 162.2 (2′-C), 193.5 (C=O).

### 4.5. Determination of Antiproliferative Activity

The antiproliferative cervical cancer activity of DMC were investigated in the human cervical cancer cell lines C-33A (ATCC^®^ HTB-31™), HeLa (ATCC^®^ CCL-2™), and SiHa (ATCC^®^ HTB-35™) using the MTT assay. The cell lines (5 × 10^3^ cells/mL) were seeded in 96-well plates and incubated for 24 h at 37 °C in 5% CO_2_ atmosphere. DMC and the three available anticancer drugs (5-fluorouracil, cisplatin, and doxorubicin) at concentrations of 0–50 µM were fixed in 1% DMSO and incubated for 48 h. The culture medium was removed and cells were washed with phosphate-buffered saline (PBS, pH 7.4). A 5 mg/mL aliquot of 3-(4,5-dimethylthiazol-2-yl)-2,5-diphenyltetrazolium bromide solution (MTT) was added, and the plate was incubated for 4 h at 37 °C in 5% CO_2_ atmosphere. The purple formazan crystals produced were dissolved in DMSO and measured using a SpectraMax i3x multi-mode microplate reader at 540 and 620 nm. The percentages of cell viability and half maximal inhibitory concentration (IC_50_) were calculated by nonlinear regression (curve fit) using GraphPad Prism version 8.0. Finally, data were analyzed for statistical significance by two-way ANOVA for active compound classification for potential anticervical cancer drugs.

### 4.6. DNA Damage Assessed by the Comet Assay

DNA damage of DMC and the available anticancer drugs was investigated. Human cervical cancer cells (HeLa) at a concentration of 2.5 × 10^5^ cells/mL were incubated in 6-well plates for 24 h at 37 °C in a 5% CO_2_ atmosphere. DMC and the available anticancer drugs (5-fluorouracil, cisplatin, and doxorubicin) were applied to HeLa cells at their IC_50_ concentration for 6, 12, 24, and 48 h. Untreated cells and cells treated with 1% (v/v) DMSO were used as the negative controls. After treatment with DMC, the cell line was investigated for DNA damage using the modified comet assay [28,29]. For cell slide preparation, glass slides were covered with 1.0% (w/v) agarose gel at 4 °C until the gel had solidified. PBS (pH 7.4) was added to the human cervical cancer cell lines (HeLa), then cell suspensions (80 µL) were fixed in low-melting agarose gel to give a final concentration of 2 × 10^4^ cells/gel. The mixture solution (330 µL) was seeded on agarose gel (1.0%) and the cover glass was applied. Then, preparation of the cell slides was performed. Cell slides were incubated overnight in lysis buffer (Tris-base 0.001 M, NaCl 2.5 M, EDTA 0.01 M, DMSO 10% (v/v), Triton-X100 1% (v/v); pH 10.0). The cell slides were neutralized with neutralizing buffer (Tris-base pH 7.5, 0.40 M) for 20 min, then DNA was separated by electrophoresis in an alkaline running buffer (Tris-base 0.90 M, NaOH 0.125 M; pH 13.0) at 20 V for 25 min. Next, the cell slides were incubated with neutralizing buffer for 20 min and stained with propidium iodide (PI; 10 µg/mL) dye for 20 min in a dark room. Cell slides were destained with deionized water (DI) three time for 20 min to remove all background PI dye. DNA damage was determined by fluorescence microscopy with a digital camera (DP70; Olympus, Tokyo, Japan) at maximum excitation and emission wavelengths of 535 and 617 nm, respectively. Data analysis was performed using Comet Score version 2.0 software. The computational parameters included the percentage tail DNA (tail DNA%) and the tail moment. Tail DNA% is the tail DNA content as a percentage of the comet DNA content. Tail moment is the tail length multiplied by the tail DNA%.

### 4.7. Cell Cycle Assay by Flow Cytometry

Cell cycle assay was performed using modified protocol from Darzynkiewicz et al., 1999; Rajamanikyam et al., 2017 and Kantapan et al., 2020 [44,45,46]. HeLa human cervical cancer cells (2.5 × 10^5^ cells) were seeded in 6-well plates and cultured for 24 h. DMC and the available anticancer drugs (5-fluorouracil, cisplatin, and doxorubicin) were applied to HeLa cells at their IC_50_ concentrations for 6, 12, 24, and 48 h. Untreated cells and cells treated with 1% (v/v) DMSO were used as the negative controls. After treatment with DMC or the anticancer drugs, human cervical cancer cells (HeLa) were fixed overnight in 70% (v/v) ethanol. Next, the cervical cancer cells were washed with PBS (pH 7.4) and centrifuged at 11,000 rpm for 1 min. PBS (pH 7.4; 11.25 µL) and Triton X-100 (0.5% v/v) were then added to the cell lines. RNase (0.5 mg/mL) and PI dye (200 ng/mL) were mixed with the human cervical cancer cells and incubated at 37 °C in a dark room for 30 min. PBS (pH 7.4; 50 µL) was then added to the cells. The DNA content, indicated by PI staining, was examined by imaging flow cytometry (FlowSight, Seattle, WA, USA) with an excitation wavelength of 595 nm and maximum emission of 642 nm. Data were analyzed using IDEAS version 6.2 (Amnis, Seattle, WA, USA).

### 4.8. Annexin V-FITC and Propidium Iodide Staining to Evaluate Apoptosis

Apoptosis assay was performed using modified protocol from Bian et al., 2019 [47]. HeLa human cervical cancer cells (2.5 × 10^5^ cells) were seeded in 6-well plates for 24 h. DMC and the available anticancer drugs (5-fluorouracil, cisplatin, and doxorubicin) were applied to HeLa cells at their IC_50_ concentrations for 24 and 48 h. Untreated cells and cells treated with 1% (v/v) DMSO were used as the negative controls. Treated and untreated HeLa cells were stained with annexin V-fluorescein isothiocyanate (FITC) using the ApopNexin Annexin V-FITC Apoptosis Kit (APT750, Millipore, Temecula, CA, USA). The fluorescence intensity of annexin V-FITC and PI staining was examined using imaging flow cytometry (FlowSight, Seattle, WA, USA). The fluorescence intensity of annexin V-FITC-positive cells was quantified at excitation and emission wavelengths of 505 and 560 nm, respectively. The fluorescence intensity of PI-stained dead cells was quantified at excitation and emission wavelengths of 595 and 642 nm, respectively. Data were analyzed using IDEAS version 6.2 (Amnis, Seattle, WA, USA).

### 4.9. Statistical Analysis

All the results were obtained in triplicate and reported as the mean ± standard deviation. Data were analyzed by two-way analysis of variance (ANOVA) using GraphPad prism version 8 statistical software. A *p*-value of ≤0.05 was considered statistically significant at the 5% significance level.

## 5. Conclusions

2′,4′-Dihydroxy-6′-methoxy-3′,5′-dimethylchalcone, a chalcone derivative that is isolated from the seed of *Syzygium nervosum* A.Cunn. ex DC. presented high antiproliferative cervical cancer activity in human cervical cancer cells (HeLa). HeLa cells treated with DMC demonstrated DNA damage, inhibition of cell proliferation, and induced apoptosis cell death.

## Figures and Tables

**Table 1:** Antiproliferative cervical cancer activities of DMC and available anticervical cancer drugs (5-fluorouracil, cisplatin, and doxorubicin) in human cervical cancer cell lines.

Compounds	Half Maximal Inhibitory Concentration ^a^ (IC_50_, µM)		
	C-33A	HeLa	SiHa
DMC	15.76 ± 1.49	10.05 ± 0.22 ^c^	18.31 ± 3.10
Doxorubicin (+) ^b^	0.08 ± 0.06	0.82 ± 0.07	0.23 ± 0.05
5-Fluorouracil (+) ^b^	88.43 ± 2.07	≥1000	205.20 ± 10.61
Cisplatin (+) ^b^	5.01 ± 0.56	9.93 ± 0.16 ^c^	9.59 ± 0.45

^a^ Expressed as the mean ± standard deviation (SD) of triplicate experiments. ^b^ Available anticervical cancer drugs. ^c^ Classification groups based on pharmacological activities in two-way ANOVA with Tukey post hoc analysis.

**Table 2:** DNA damage parameters of HeLa human cervical cancer cells after treatment with DMC and available anticervical cancer drugs, assessed using the comet assay.

Incubation Time (h)	Sample	DNA Damage Parameters ^a^					
		Tail DNA%	Comparison (Fold Change)		Tail Moment	Comparison (Fold Change)	
			Untreated	1% DMSO		Untreated	1% DMSO
6	Untreated	4.61 ± 1.62	-	2	0.52 ± 0.63	-	4
	1% DMSO	2.38 ± 1.63	1	-	0.14 ± 0.16	0	-
	DMC	65.07 ± 9.57	14	27	53.29 ± 5.21	102	381
	Doxorubicin	56.61 ± 3.09	12	24	45.49 ± 4.65	87	325
	5-Fluorouracil	51.33 ± 12.29	11	22	39.33 ± 11.53	76	281
	Cisplatin	45.00 ± 11.71	10	19	42.50 ± 10.38	82	304
12	Untreated	3.33 ± 2.66	-	1	0.32 ± 0.43	-	1
	1% DMSO	4.43 ± 0.63	1	-	0.24 ± 0.10	1	-
	DMC	49.98 ± 8.78	15	11	34.37 ± 5.31	107	143
	Doxorubicin	52.37 ± 3.03	16	12	38.84 ± 3.25	121	162
	5-Fluorouracil	44.07 ± 8.13	13	10	28.20 ± 9.70	88	118
	Cisplatin	40.40 ± 7.74	12	9	31.45 ± 1.67	98	131
24	Untreated	4.44 ± 0.86	-	1	0.25 ± 0.12	-	1
	1% DMSO	4.16 ± 1.42	1	-	0.22 ± 0.20	1	-
	DMC	76.55 ± 6.00	17	18	78.13 ± 11.41	313	355
	Doxorubicin	78.39 ± 1.64	18	19	60.12 ± 9.20	240	273
	5-Fluorouracil	36.08 ± 11.23	8	9	22.69 ± 10.96	91	103
	Cisplatin	41.40 ± 1.49	9	10	30.96 ± 7.40	124	141
48 h	Untreated	3.67 ±0.67	-	1	0.14 ± 0.03	-	2
	1% DMSO	3.06 ± 0.48	1	-	0.09 ± 0.04	1	-
	DMC	92.17 ± 1.76	25	30	116.65 ± 9.93	833	1296
	Doxorubicin	86.13 ± 5.81	23	28	72.92 ± 1.91	521	810
	5-Fluorouracil	37.32 ± 9.20	10	12	20.09 ± 5.30	144	223
	Cisplatin	29.71 ± 7.51	8	10	16.56 ± 2.57	118	184

^a^ Data are expressed as the mean ± standard deviation (SD) of triplicate experiments.

## Data Availability

Not applicable.

## Supplementary Materials

The following are available online. Figure S1: TLC Chromatogram of crude CH_2_Cl_2_ extract from *S. nervosum* seeds (left) compared with standard DMC (right) visualized under UV 254 nm, Figure S2: UV–VIS spectrum of DMC, Figure S3: FT-IR (ATR) spectrum of DMC, Figure S4: ^1^H-NMR (CDCl_3_, 500 MHz) spectrum of DMC, Figure S5: ^13^C-NMR (CDCl_3_, 125 MHz) combined with DEPT90 and DEPT135 spectra of DMC, Figure S6: ^1^H-^1^H COSY spectrum of DMC, Figure S7: ^1^H-^13^C HSQC spectrum of DMC, Figure S8: ^1^H-^13^C HMBC spectrum of DMC, Figure S9: Cell cycle of Hela treated with DMC by flow cytometry, Table S1: ^1^H-NMR (500 MHz) and ^13^C-NMR (125 MHz) spectra in CDCl_3_ for DMC compared with literature [15].

## Sample Availability

Samples of the compounds are available from the authors.

## References

[1] Bray F., Ferlay J., Soerjomataram I., Siegel R.L., Torre L.A., Jemal A. (2018). Global cancer statistics 2018: GLOBOCAN estimates of incidence and mortality worldwide for 36 cancers in 185 countries. CA Cancer J. Clin..

[2] Ferlay J., Colombet M., Soerjomataram I., Mathers C., Parkin D.M., Piñeros M., Znaor A., Bray F. (2019). Estimating the global cancer incidence and mortality in 2018: GLOBOCAN sources and methods. Int. J. Cancer.

[3] Siegel R.L., Miller K.D., Jemal A. (2020). Cancer statistics, 2020. CA Cancer J. Clin..

[4] Wilailak S., Kengsakul M., Kehoe S. (2021). Worldwide initiatives to eliminate cervical cancer. Int. J. Gynecol. Obstet..

[5] Bhatla N., Aoki D., Sharma D.N., Sankaranarayanan R. (2018). Cancer of the cervix uteri. Int. J. Gynecol. Obstet..

[6] Marth C., Landoni F., Mahner S., McCormack M., Gonzalez-Martin A., Colombo N., ESMO Guidelines Committee (2017). Cervical cancer: ESMO Clinical Practice Guidelines for diagnosis, treatment and follow-up. Ann. Oncol..

[7] Han R., Yang Y.M., Dietrich J., Luebke A., Mayer-Pröschel M., Noble M. (2008). Systemic 5-fluorouracil treatment causes a syn-drome of delayed myelin destruction in the central nervous system. J. Biol..

[8] Astolfi L., Ghiselli S., Guaran V., Chicca M., Simoni E., Olivetto E., Lelli G., Martini A. (2013). Correlation of adverse effects of cisplatin administration in patients affected by solid tumours: A retrospective evaluation. Oncol. Rep..

[9] Zhao N., Woodle M.C., Mixson A.J. (2018). Advances in Delivery Systems for Doxorubicin. J. Nanomed. Nanotechnol..

[10] Pham G.N., Nguyen T.T.T., Nguyen-Ngoc H. (2020). Ethnopharmacology, phytochemistry, and pharmacology of *Syzygium ner-vosum*. Evidence-Based Complement. Altern. Med..

[11] Inboot W., Taya S., Chailungka A., Meepowpan P., Wongpoomchai R. (2012). Genotoxicity and antigenotoxicity of the methanol extract of *Cleistocalyx nervosum* var. paniala seed using a *Salmonella* mutation assay and rat liver micronucleus tests. Mol. Cell. Toxicol..

[12] Yu W.-G., He H., Qian J., Lu Y.-H. (2014). Dual Role of 2′,4′-Dihydroxy-6′-methoxy-3′,5′-dimethylchalcone in inhibiting high-mobility group box 1 secretion and blocking its pro-inflammatory activity in hepatic inflammation. J. Agric. Food Chem..

[13] Wu J.-H., Wang X.-H., Yi Y.-H., Lee K.-H. (2003). Anti-AIDS agents 54. A potent anti-HIV chalcone and flavonoids from genus Desmos. Bioorg. Med. Chem. Lett..

[14] Luo Y., Lu Y. (2012). 2′,4′-Dihydroxy-6′-methoxy-3′,5′-dimethylchalcone inhibits apoptosis of MIN6 cells via improving mitochon-drial function. Pharmazie.

[15] Chailungka A., Junpirom T., Pompimon W., Nuntasaen N., Meepowpan P. (2017). Two flavonoids first isolated from the seed of *Syzygium nervosum* and preliminary study of their anticancer and anti-HIV-1 reverse transcriptase activities. Maejo Int. J. Sci. Technol..

[16] Khamto N., Chaichuang L., Rithchumpon P., Phupong W., Bhoopong P., Tateing S., Pompimon W., Semakul N., Chomsri N., Meepowpan P. (2021). Synthesis, cytotoxicity evaluation and molecular docking studies on 2′,4′-Dihydroxy-6′-methoxy-3′,5′-dimethylchalcone derivatives. RSC Adv..

[17] Santana-Méridas O., González-Coloma A., Sánchez-Vioque R. (2012). Agricultural residues as a source of bioactive natural products. Phytochem. Rev..

[18] Srivastava R., Shaw A.K., Kulshreshtha D.K. (1995). Triterpenoids and chalcone from *Syzygium samarangense*. Phytochemistry.

[19] Amor E.C., Villaseñor I.M., Yasin A., Choudhary M.I. (2004). Prolyl endopeptidase inhibitors from *Syzygium samarangense* (Blume) Merr. & L. M. Perry. Z. Naturforsch. C.

[20] Hadisaputri Y.E., Cahyana N., Muchtaridi M., Lesmana R., Rusdiana T., Chaerunisa A.Y., Sufiawati I., Rostinawati T., Subarnas A. (2020). Apoptosis-mediated antiproliferation of A549 lung cancer cells mediated by Eugenia aquea leaf compound 2′,4′-Dihydroxy-6′-methoxy-3′,5′-dimethylchalcone and its molecular interaction with caspase receptor in molecular docking simulation. Oncol. Lett..

[21] Ye C.-L., Qian F., Wei D.-Z., Lu Y.-H., Liu J.-W. (2005). Induction of apoptosis in K562 human leukemia cells by 2′,4′-Dihydroxy-6′-methoxy-3′,5′-dimethylchalcone. Leuk. Res..

[22] Ko H., Kim Y.-J., Amor E.C., Lee J.W., Kim H.-C., Kim H.J., Yang H.O. (2011). Induction of autophagy by dimethyl cardamonin is associated with proliferative arrest in human colorectal carcinoma HCT116 and LOVO cells. J. Cell. Biochem..

[23] Tuan H.N., Minh B.H., Tran P.T., Lee J.H., Van Oanh H., Ngo Q.M.T., Nguyen Y.N., Lien P.T.K., Tran M.H. (2019). The Effects of 2′,4′-Dihydroxy-6′-methoxy-3′,5′-dimethylchalcone from *Cleistocalyx operculatus* buds on human pancreatic cancer cell lines. Molecules.

[24] Ye C.-L., Liu J.-W., Wei D.-Z., Lu Y.-H., Qian F. (2005). In vivo antitumor activity by 2′,4′-Dihydroxy-6′-methoxy-3′,5′-dimethylchalcone in a solid human carcinoma xenograft model. Cancer Chemother. Pharmacol..

[25] Ye C.-L., Lai Y.-F. (2016). 2′,4′-Dihydroxy-6′-methoxy-3′,5′-dimethylchalcone, from buds of *Cleistocalyx operculatus*, induces apop-tosis in human hepatoma SMMC-7721 cells through a reactive oxygen species-dependent mechanism. Cytotechnology.

[26] Rammohan A., Reddy J.S., Sravya G., Rao C.N., Zyryanov G.V. (2020). Chalcone synthesis, properties and medicinal applications: A review. Environ. Chem. Lett..

[27] Subarnas A., Diantini A., Abdulah R., Zuhrotun A., Hadisaputri E., Puspitasari M.Y., Yamazaki C.I., Kuwano H., Koyama H. (2015). Apoptosis induced in MCF-7 human breast cancer cells by 2′,4′-Dihydroxy-6′-methoxy-3′,5′-dimethylchalcone isolated from *Eugenia aquea Burm f*. leaves. Oncol. Lett..

[28] Collins A.R. (2004). The Comet Assay for DNA Damage and Repair: Principles, Applications, and Limitations. Mol. Biotechnol..

[29] Olive P.L., Banáth J.P. (2006). The comet assay: A method to measure DNA damage in individual cells. Nat. Protoc..

[30] Speit G., Hartmann A. (2005). The Comet Assay: A Sensitive Genotoxicity Test for the Detection of DNA Damage. Methods Mol. Biol..

[31] Jagetia A., Jagetia G.C., Jha S. (2006). Naringin, a grapefruit flavanone, protects V79 cells against the bleomycin-induced genotoxicity and decline in survival. J. Appl. Toxicol..

[32] Dao T.-T., Tung B.-T., Nguyen P.-H., Thuong P.-T., Yoo S.-S., Kim E.-H., Kim S.-K., Oh W.-K. (2010). C-Methylated Flavonoids from *Cleistocalyx operculatus* and Their Inhibitory Effects on Novel Influenza A (H1N1) Neuraminidase. J. Nat. Prod..

[33] Yang Y., Jiang G., Zhang P., Fan J. (2015). Programmed cell death and its role in inflammation. Mil. Med. Res..

[34] Wang C., Wu P., Shen X.-L., Wei X.-Y., Jiang Z.-H. (2017). Synthesis, cytotoxic activity and drug combination study of tertiary amine derivatives of 2′,4′-Dihydroxy-6′-methoxy-3′,5′-dimethylchalcone. RSC Adv..

[35] Deans A.J., West S.C. (2011). DNA interstrand crosslink repair and cancer. Nat. Rev. Cancer.

[36] He G., Siddik Z.H., Huang Z., Wang R., Koomen J., Kobayashi R., Khokhar A.R., Kuang J. (2005). Induction of p21 by p53 fol-lowing DNA damage inhibits both Cdk4 and Cdk2 activities. Oncogene.

[37] Zhang L., Yang X., Li X., Li C., Zhao L., Zhou Y., Hou H. (2015). Butein sensitizes HeLa cells to cisplatin through the AKT and ERK/p38 MAPK pathways by targeting FoxO_3_a. Int. J. Mol. Med..

[38] Kciuk M., Bukowski K., Marciniak B., Kontek R. (2020). Advances in DNA Repair—Emerging Players in the Arena of Eukaryotic DNA Repair. Int. J. Mol. Sci..

[39] Jain A., Jahagirdar D., Nilendu P., Sharma N.K. (2017). Molecular approaches to potentiate cisplatin responsiveness in carcinoma therapeutics. Expert Rev. Anticancer Ther..

[40] Mann J.R., Backlund M.G., Dubois R.N. (2005). Mechanisms of Disease: Inflammatory mediators and cancer prevention. Nat. Clin. Pract. Oncol..

[41] Norbury C.J., Zhivotovsky B. (2004). DNA damage-induced apoptosis. Oncogene.

[42] Erasimus H., Gobin M., Niclou S., Van Dyck E. (2016). DNA repair mechanisms and their clinical impact in glioblastoma. Mutat. Res. Mutat. Res..

[43] Ngabire D., Seong Y.-A., Patil M.P., Niyonizigiye I., Seo Y.B., Kim G.-D. (2018). Induction of apoptosis and G1 phase cell cycle arrest by *Aster incisus* in AGS gastric adenocarcinoma cells. Int. J. Oncol..

[44] Darzynkiewicz Z., Juan G., Bedner E. (1999). Determining Cell Cycle Stages by Flow Cytometry. Curr. Protoc. Cell Biol..

[45] Rajamanikyam M., Vadlapudi V., Parvathaneni S.P., Koude D., Sripadi P., Misra S., Amanchy R., Upadhyayula S.M. (2017). Isolation and characterization of phthalates from *Brevibacterium mcbrellneri* that cause cytotoxicity and cell cycle arrest. EXCLI J..

[46] Kantapan J., Paksee S., Chawapun P., Sangthong P., Dechsupa N. (2020). Pentagalloyl Glucose- and Ethyl Gallate-Rich Extract from Maprang Seeds Induce Apoptosis in MCF-7 Breast Cancer Cells through Mitochondria-Mediated Pathway. Evid.-Based Complement. Altern. Med..

[47] Bian S., Zhao Y., Li F., Lu S., Wang S., Bai X., Liu M., Zhao D., Wang J., Guo D. (2019). 20(S)-Ginsenoside Rg3 Promotes HeLa Cell Apoptosis by Regulating Autophagy. Molecules.

