# Supplementary material for: Effects of 2′,4′-Dihydroxy-6′-methoxy-3′,5′-dimethylchalcone from Syzygium nervosum Seeds on Antiproliferative, DNA Damage, Cell Cycle Arrest, and Apoptosis in Human Cervical Cancer Cell Lines
Source: Molecules. 2022 Feb 9;27(4):1154. doi: 10.3390/molecules27041154 (PMC8879438; doi:10.3390/molecules27041154)
Supplement: Supplementary file 1 [file molecules-27-01154-s001.zip › molecules-1554366-supplementary.pdf]

Supplement data

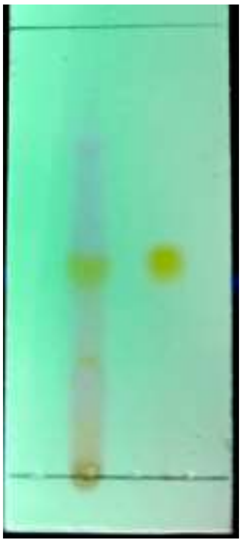

Figure S1. TLC Chromatogram of crude CH_2Cl_2 extract from *S. nervosum* seeds (left) compared with standard DMC (right) visualized under UV 254 nm

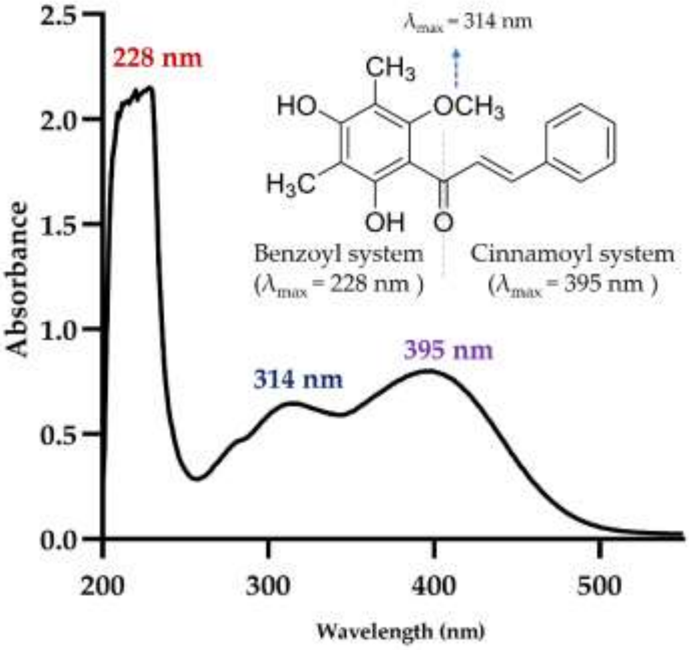

Figure S2. UV-VIS spectrum of 2′,4′-dihydroxy-6′-methoxy-3′,5′-dimethylchalcone (DMC)

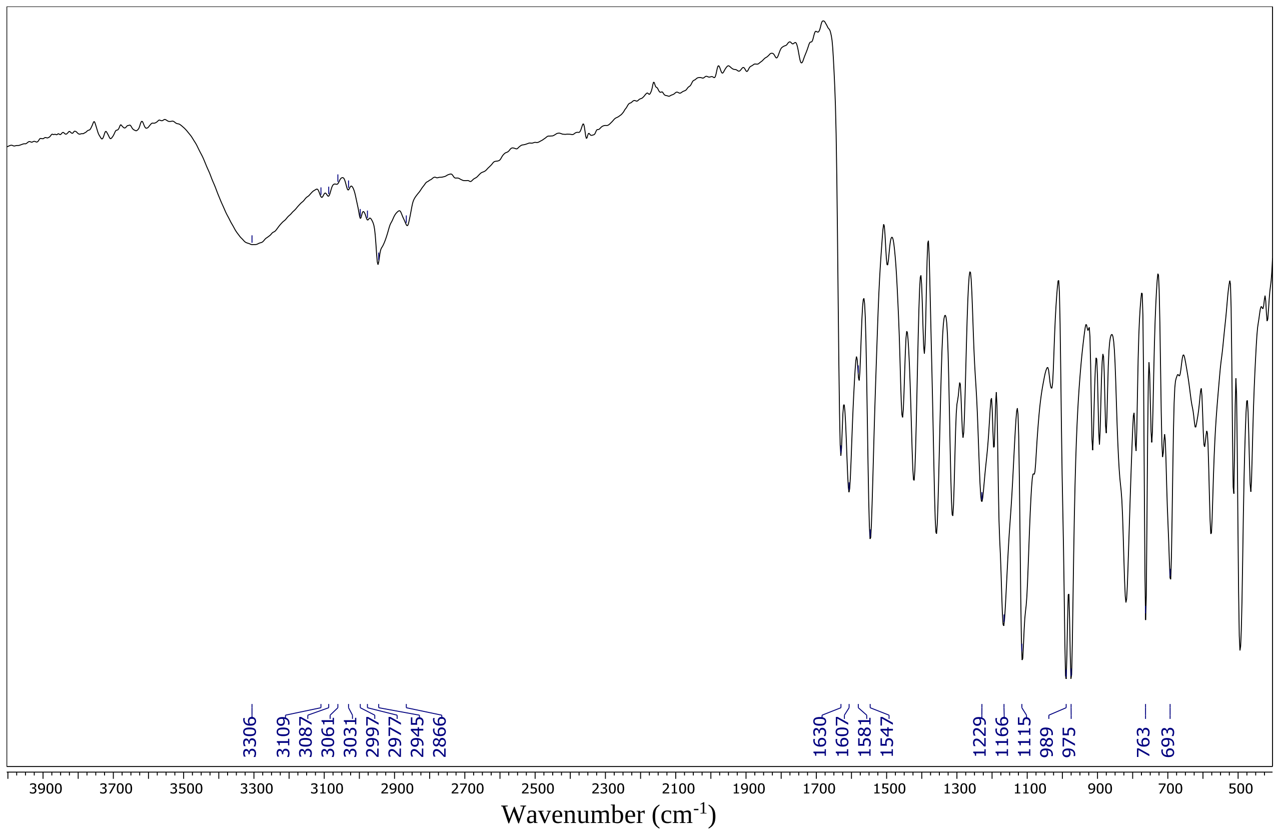

Figure S3. FT-IR (ATR) spectrum of 2′,4′-dihydroxy-6′-methoxy-3′,5′-dimethylchalcone (DMC)

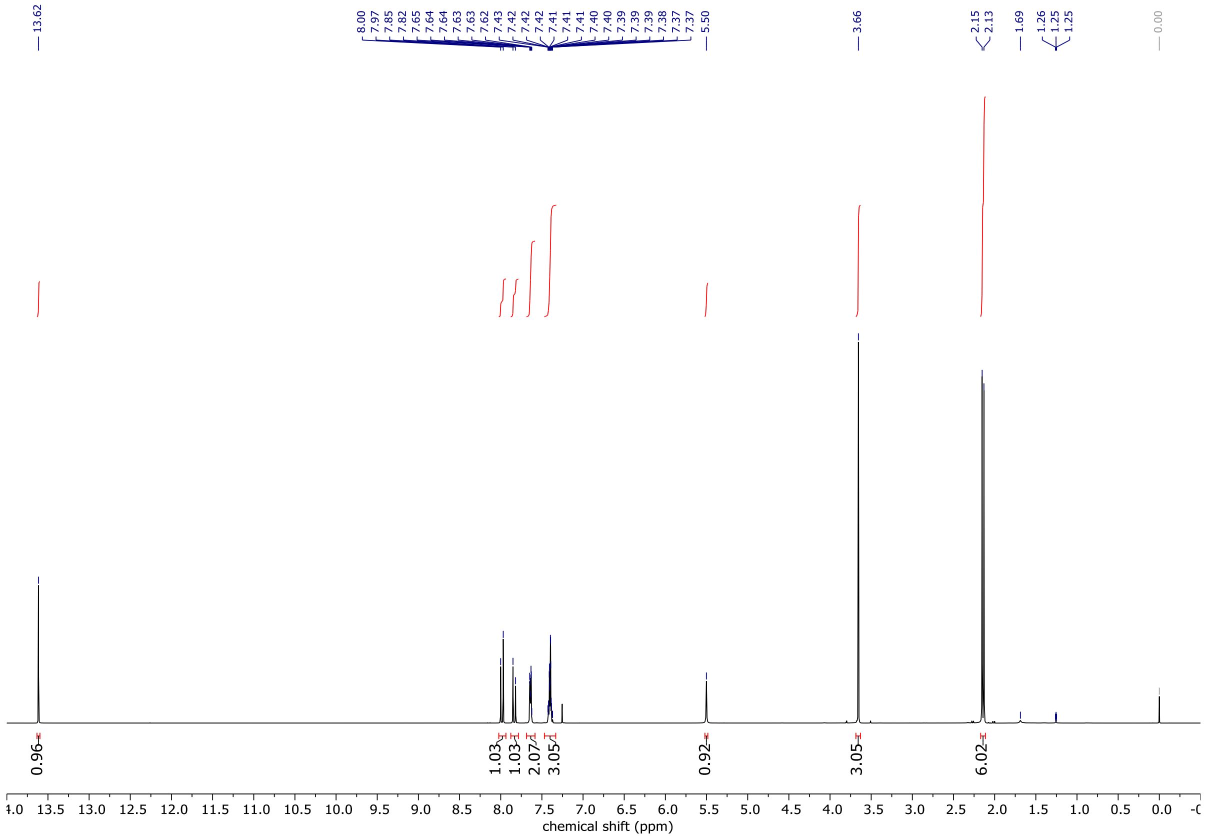

Figure S4. ^{1}H-NMR ($CDCl_3$, 500 MHz) spectrum of 2′,4′-dihydroxy-6′-methoxy-3′,5′-dimethylchalcone (DMC)

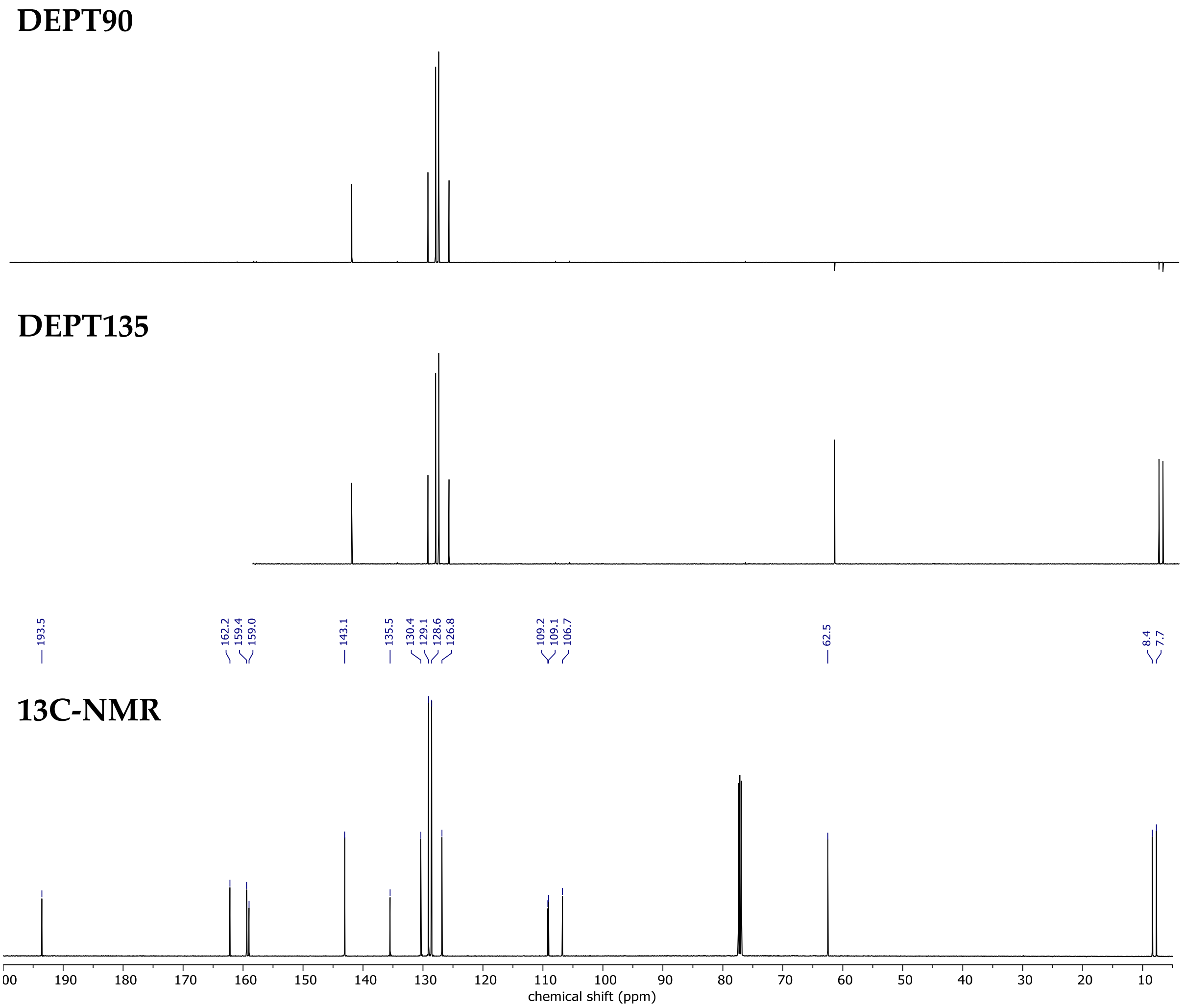

Figure S5. ^{13}C-NMR ($CDCl_3$, 125 MHz) combined with DEPT90 and DEPT135 spectra of 2′,4′-dihydroxy-6′-methoxy-3′,5′-dimethylchalcone (DMC)

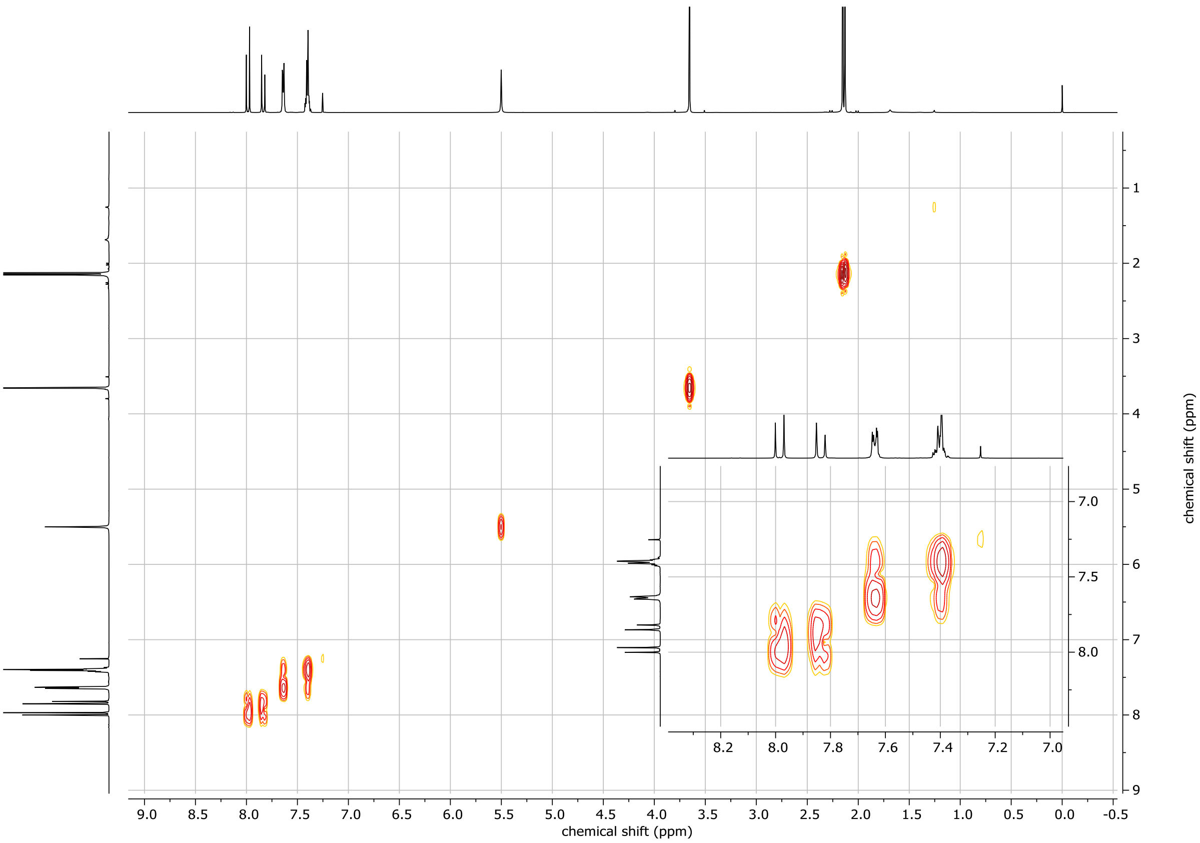

Figure S6 ^{1}H-^{1}H COSY spectrum of 2′,4′-dihydroxy-6′-methoxy-3′,5′-dimethylchalcone (DMC)

The complete chemical structure of DMC was confirmed by 2D-NMR methods including ^{1}H-^{1}H Correlation spectroscopy (COSY), heteronuclear single-quantum coherence (HSQC) and heteronuclear multiple-bond correlation (HMBC). COSY experiment illustrated the correlation of the neighbouring protons which indicated the correlation between α,β-unsaturated protons at δ 7.99 (d, J = 15.7 Hz, β-CH) and 7.84 (d, J = 15.7 Hz, α-CH) ppm. Moreover, the correlation between aromatic protons were also observed at δ 7.37–7.44 (m, ArCH), 7.61–7.67 (m, ArCH).

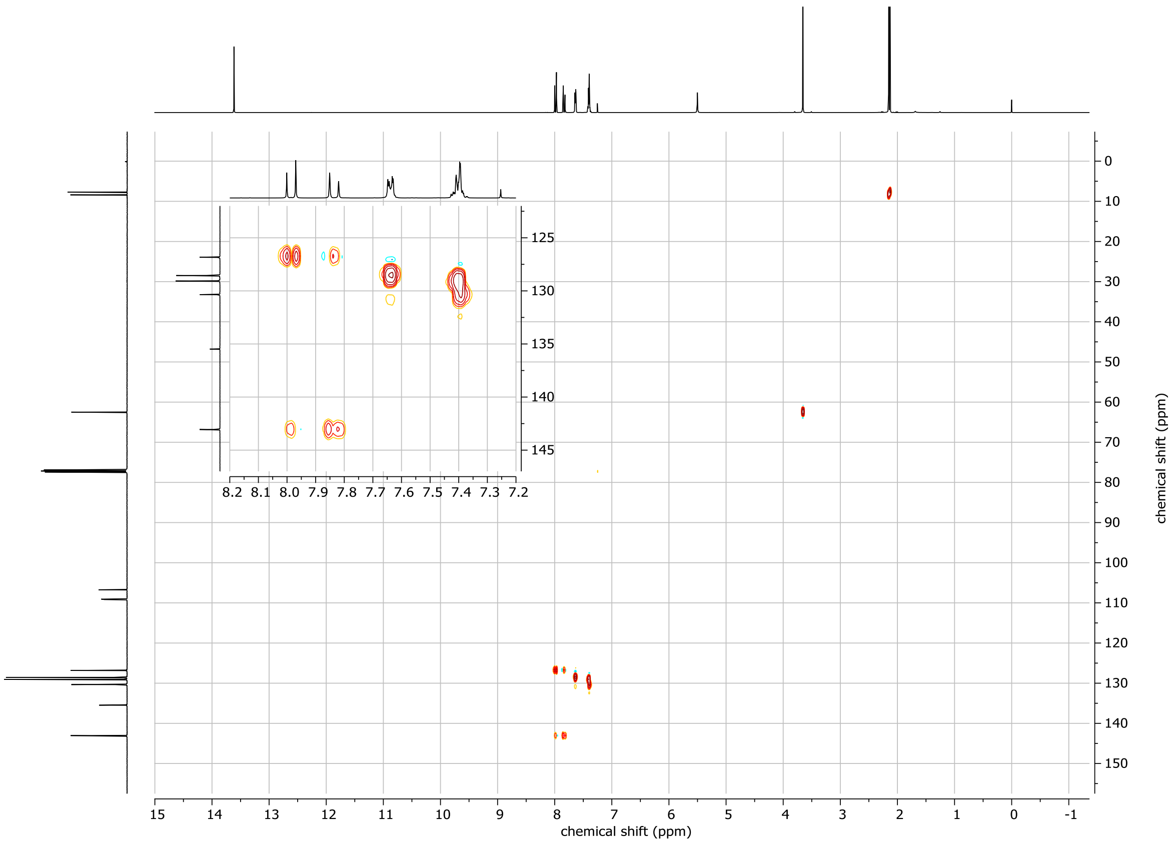

Figure S7 ^{1}H-^{13}C HSQC spectrum of 2′,4′-dihydroxy-6′-methoxy-3′,5′-dimethylchalcone (DMC)

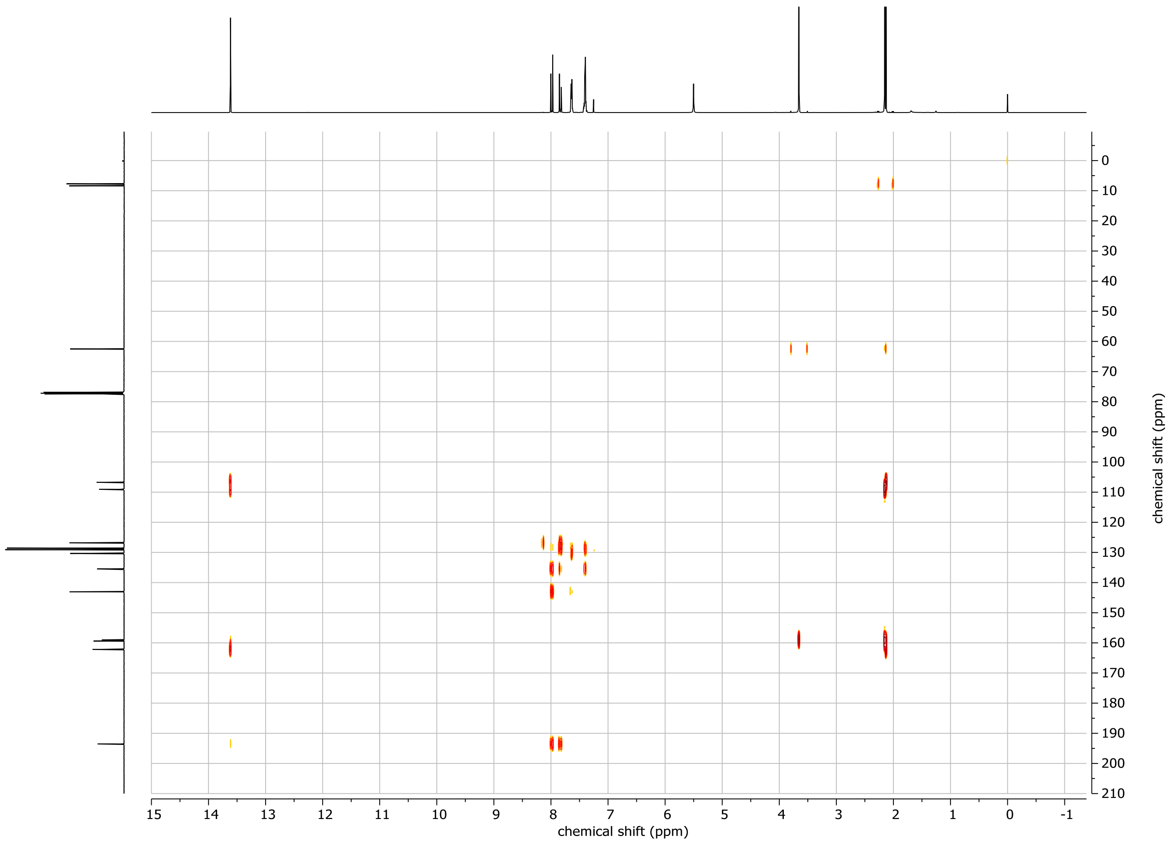

Figure S8 ^{1}H-^{13}C HMBC spectrum of 2′,4′-dihydroxy-6′-methoxy-3′,5′-dimethylchalcone (DMC)

HSQC experiment allows us to clarify the connection between protons and carbons. HMBC experiment also used for elucidation of the connection between protons and neighbouring carbons. The HMBC spectrum indicated the correlation of β-CH at δ 7.84 ppm with carbon signals at δ 135.3 (1-C), 128.9 (2,6-C), 126.7 (α-CH), and 193.4 (C=O). The α-CH resonating at δ 7.99 ppm showed correlation with carbon atoms of 135.3 (1-C), 142.9 (β-CH), 193.4 (C=O) and 106.6 (1′-C) ppm. The proton's signals of 3′-CH_3 resonating at δ 2.14 ppm correlated with the carbon atom signals at δ 162.0 (2′-C), 109.0 (3′-C), 159.3 (4′-C). In addition, the signal of 5′-CH_3 demonstrated correlation with carbon atom signals at δ 159.3 (4′-C), 109.0 (5′-C) and 158.8 (6′-C). The methoxy group protons of 6′-OCH_3 resonating at δ 3.66 ppm correlated with the carbon signal at 158.8 (6′-OCH_3) ppm.

Table S1 ^{1}H-NMR (500 MHz) and ^{13}C-NMR (125 MHz) spectra in $CDCl_3$ for DMC compared with literature [15].

**Position**	δ ^{1}H (*J* in Hz)* (experiment)	δ ^{1}H (*J* in Hz)* (literature)	δ ^{13}C (DEPT) (experiment)	δ ^{13}C (DEPT) (literature)
1	-	-	135.5 (C)	135.3 (C)
2	7.61–7.67 *m*	7.64 *m*	129.1 (CH)	128.9 (CH)
3	7.37–7.44 *m*	7.41 *m*	128.6 (CH)	128.4 (CH)
4	7.37–7.44 *m*	7.41 *m*	130.4 (CH)	130.2 (CH)
5	7.37–7.44 *m*	7.41 *m*	128.6 (CH)	128.4 (CH)
6	7.61–7.67 *m*	7.64 *m*	129.1 (CH)	128.9 (CH)
α	7.84 *d* (15.7)	7.99 *d* (15.7)	126.8 (CH)	126.7 (CH)
β	7.99 *d* (15.7)	7.84 *d* (15.7)	143.1 (CH)	142.9 (CH)
C=O	-	-	193.5 (C)	193.4 (C)
1′	-	-	106.7 (C)	106.6 (C)
2′	-	-	162.2 (C)	162.0 (C)
3′	-	-	109.1 (C)	109.0 (C)
4′	-	-	159.4 (C)	159.3 (C)
5′	-	-	109.2 (C)	109.0 (C)
6′	-	-	159.0 (C)	158.8 (C)
2′-OH	13.62 *s*	13.69 *s*	-	-
3′-CH_3	2.13 *s*	2.14 *s*	8.4 (CH_3)	8.2 (CH_3)
4′-OH	5.50 *s*	5.38 *s*	-	-
5′-CH_3	2.15 *s*	2.16 *s*	7.7 (CH_3)	7.6 (CH_3)
6′-OCH_3	3.66 *s*	3.66 *s*	62.5 (CH_3)	62.3 (CH_3)

* Chemical shift (δ) in ppm deshielded from TMS [coupling constant (*J*) in Hz are given in parentheses]

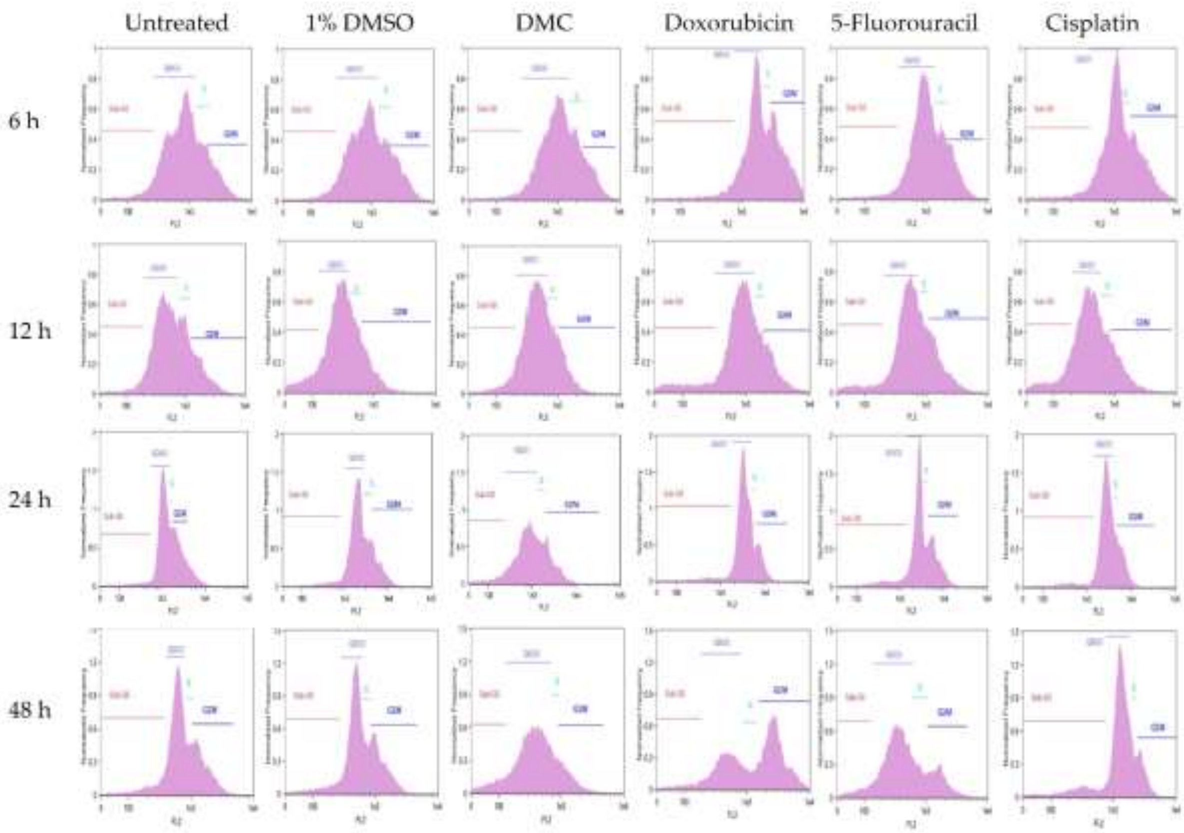

Figure S9 Cell cycle of HeLa treated with DMC, Doxorubicin, 5-Fluorouracil and Cisplatin by flow cytometry
